# Notch and Bmp signaling pathways act coordinately during the formation of the proepicardium

**DOI:** 10.1002/dvdy.229

**Published:** 2020-09-03

**Authors:** Laura Andrés‐Delgado, María Galardi‐Castilla, Juliane Münch, Marina Peralta, Alexander Ernst, Juan Manuel González‐Rosa, Federico Tessadori, Luis Santamaría, Jeroen Bakkers, Julien Vermot, José Luis de la Pompa, Nadia Mercader

**Affiliations:** ^1^ Development of the Epicardium and its Role During Regeneration Laboratory National Center of Cardiovascular Research Carlos III Madrid Spain; ^2^ Department of Anatomy, Histology, and Neuroscience, School of Medicine Autonoma University of Madrid Madrid Spain; ^3^ Intercellular Signaling in Cardiovascular Development and Disease Laboratory National Center of Cardiovascular Research Carlos III Madrid Spain; ^4^ Ciber CV Madrid Spain; ^5^ Institute of Biochemistry and Biology Potsdam University Potsdam Germany; ^6^ Institute of Genetics and Molecular and Cellular Biology (IGBMC) Illkirch France; ^7^ Australian Regenerative Institute Monash University Clayton Victoria Australia; ^8^ Institute of Anatomy University of Bern Bern Switzerland; ^9^ Cardiovascular Research Center, Massachusetts General Hospital and Harvard Medical School Boston Massachusetts USA; ^10^ Hubrecht Institute‐KNAW and UMC Utrecht Utrecht The Netherlands; ^11^ Division of Heart and Lungs, Department of Medical Physiology University Medical Center Utrecht The Netherlands; ^12^ Department of Bioengineering Imperial College London London UK

**Keywords:** heart development, pericardium, zebrafish

## Abstract

**Background:**

The epicardium is the outer mesothelial layer of the heart. It encloses the myocardium and plays key roles in heart development and regeneration. It derives from the proepicardium (PE), cell clusters that appear in the dorsal pericardium (DP) close to the atrioventricular canal and the venous pole of the heart, and are released into the pericardial cavity. PE cells are advected around the beating heart until they attach to the myocardium. Bmp and Notch signaling influence PE formation, but it is unclear how both signaling pathways interact during this process in the zebrafish.

**Results:**

Here, we show that the developing PE is influenced by Notch signaling derived from the endothelium. Overexpression of the intracellular receptor of *notch* in the endothelium enhances *bmp* expression, increases the number of pSmad1/5 positive cells in the DP and PE, and enhances PE formation. On the contrary, pharmacological inhibition of Notch1 impairs PE formation. *bmp2b* overexpression can rescue loss of PE formation in the presence of a Notch1 inhibitor, but Notch gain‐of‐function could not recover PE formation in the absence of Bmp signaling.

**Conclusions:**

Endothelial Notch signaling activates *bmp* expression in the heart tube, which in turn induces PE cluster formation from the DP layer.

## Key Findings

- Notch is active on endothelial cells but not on EPDCs cells during PE formation.
- Endothelial Notch signaling activates *Bmp* expression in the heart‐tube.
- Notch and Bmp pathways induce PE cluster formation from the DP layer.

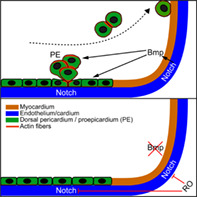

## 1 INTRODUCTION

The epicardium is the mesothelial layer of the heart that covers the myocardium. During developmental stages, the epicardium protects and nurtures the underlying myocardium through paracrine signals that promote its growth.^1^, ^2^ The embryonic epicardium also acts as a source of epicardial‐derived cells (EPDCs), that can differentiate into other cell types^3^ such as adipocytes,^4^, ^5^, ^6^ vascular smooth muscle cells and cardiac fibroblasts.^7^, ^8^, ^9^ EPDCs are also involved in several aspects of tissue repair and regeneration after an injury. For example, they contribute to cardiac fibrosis, control the inflammatory response and promote neoangiogenesis and proliferation of cardiomyocytes.^10^

The epicardium arises from an extracardiac structure called the proepicardium (PE). The PE is formed by clusters of cells that derive from the dorsal pericardial mesothelium (DP). It develops close to the venous pole of the heart tube, around the time of heart looping, and after the onset of heart beating.^11^, ^12^ In zebrafish, the PE forms by a mechanism that involves the constriction of the DP layer and the extrusion of PE cells to the pericardial cavity.^13^ Once the PE clusters form, the pericardial fluid flow generated by the heartbeat allows PE cells to detach and reach the myocardium. PE cells spreads on top myocardium to form a new tissue layer: the epicardium.^14^, ^15^

Little is known about the molecular mechanisms involved in PE formation. In zebrafish, Bmp signaling is essential for PE specification. Animals lacking the Bmp receptor Acvr1l fail to form a PE, whereas *bmp2b* overexpression extends PE marker gene expression.^16^ The Bmp pathway affects actomyosin cytoskeleton rearrangements and promotes the constriction of the DP during the generation of the PE.^13^ Bmp2 is also important for the generation of PE extrusion and the adhesion to the myocardial surface in the chicken.^17^ In human induced pluripotent stem cell cultures, temporally controlled activity of Bmp is necessary for the differentiation into an epicardal cell fate.^18^, ^19^

Another crucial regulator of cardiovascular development is the NOTCH pathway. NOTCH signaling controls numerous processes including endocardial cushion formation, proliferation of the endothelium, maturation of the myocardium, arterial‐venous fate decisions and angiogenesis.^20^, ^21^ Expression of *Notch1* in mesothelial EPDCs in the forming chick heart suggested a role of the NOTCH pathway in the developing epicardium.^22^ Moreover, loss of NOTCH function alters epicardium formation, and NOTCH signaling regulates smooth muscle differentiation of epicardium‐derived cells.^23^, ^24^ A relationship between NOTCH and BMP2 pathways during cardiovascular development was reported in the mouse. On the one hand, ectopic NOTCH1 activation in the myocardium expands the expression of its effector *Hey1* to non‐chamber myocardium, which represses BMP2 and disrupts valve tissue specification and epithelial to mesenchymal transition (EMT). On the other hand, conditional *BMP2* inactivation in the myocardium impairs NOTCH1 activity, suggesting a functional link between these two signaling pathways.^25^ In endocardial cells NOTCH1 signaling induces the expression of *Wnt4*, which upregulates *Bmp2* expression in the adjacent atrioventricular canal myocardium.^26^ Also in mice, NOTCH signaling within the PE inhibits BMP2 signaling while myocardial BMP2 activity induces endocardial JAG1‐NOTCH signaling.^23^ It is unknown if the molecular mechanisms that lead to PE formation are conserved in the zebrafish.

Here, we investigated the role of Notch1 signaling during zebrafish PE formation. We found that Notch signaling inhibition impairs PE formation. Overactivation of Notch signaling in EPDCs did not lead to any alteration, but when overexpressed in the endothelium, PE formation was enhanced. We found that in the zebrafish, the effect of Notch activity on the PE was at least partially dependent on its role in promoting *bmp2/4* expression, which differs from mice. Thus, we describe a signaling relay mechanism through different tissues in which endothelial/endocardial Notch signaling augments *bmp* expression in the heart tube, ultimately promoting Bmp signaling in mesothelial cells forming PE clusters.

## 2 RESULTS

### 2.1 Endothelial Notch signaling promotes proepicardium formation

**FIGURE 1:**
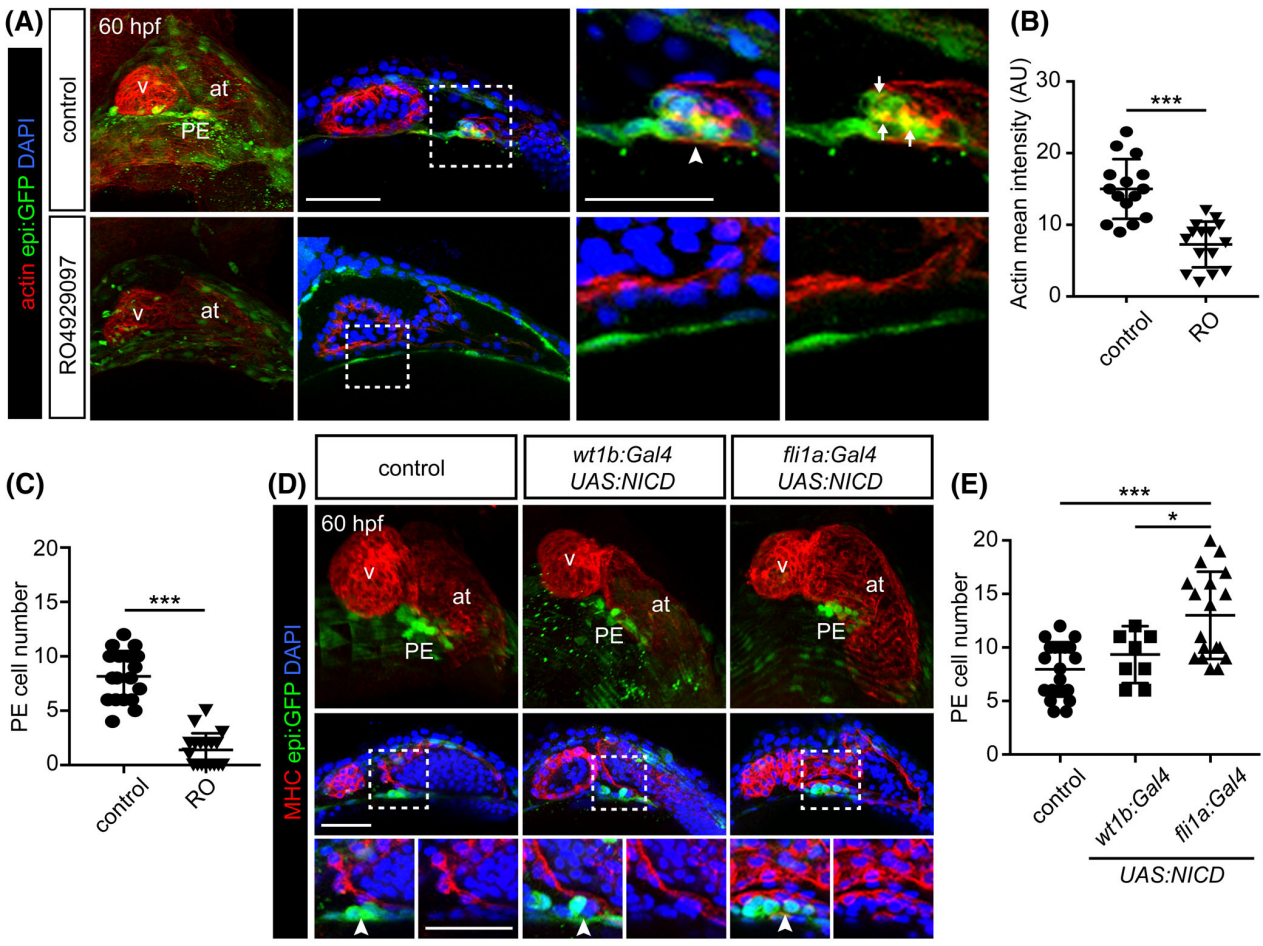
Notch signaling in the endothelium is necessary for proepicardium formation. A, 3D projections, optical sections and zoomed images of a 60 hpf control zebrafish heart compared with a RO‐treated animal. *epi:GFP* animals immunostained for GFP (green), F‐actin is detected with fluorescently‐labeled phalloidin (red) and nuclei counterstained with DAPI (blue). Arrowhead, PE cluster. Arrows, accumulation of F‐actin in the PE. B, Quantification of actin intensity (arbitrary units) in PE cells from conditions shown in A. C, Quantification of PE cell number in A. D, Top panels, 3D projection of a 60 hpf zebrafish heart, middle panels optical section and zoomed images below. The DP was digitally isolated in 3D projections. Control compared to those overexpressing *NICD* in pericardial and proepicardial cells (*wt1b:Gal4*) or in endothelial cells (*fli1a:Gal4*). *epi:GFP* embryos immunostained for GFP (green), myosin heavy chain (MHC, red) and nuclei counterstained with DAPI (blue). Arrowheads, PE cluster. E, Quantification of PE cell number in D. at, atrium; DP, dorsal pericardium; hpf; hours post fertilization; PE, proepicardium; v, ventricle. Scale bar: 50 μm. Data are means ± SD. Unpaired two‐tailed Student's *t*‐test in B and C. One‐way ANOVA followed by Kruskal‐Wallis significant difference test in E. *P < .05, ***P < .001

To determine whether Notch signaling regulates PE formation in the zebrafish, we treated embryos with the Notch inhibitor RO4929097 (RO).^27^ To label the DP and PE we used the enhancer trap line *Et(−26.5Hsa.WT1‐gata2:EGFP)* ^*cn1*^ (hereafter *epi:GFP*)^14^ in which GFP expression is controlled by the *wilms tumor 1a* (*wt1a*) regulatory elements, and recapitulates *wt1a* expression pattern. Previously, we have illustrated the importance of actin dynamics for PE formation.^13^ Here, we observed that upon abrogation of Notch activity, F‐actin was significantly decreased at 60 hr post fertilization (hpf) (Figure 1A,B). We also found that RO administration from 48 hpf onwards impaired PE formation by reducing the PE cluster size at 60 hpf (2 ± 2 cells vs. 8 ± 3 cells in controls) (Figure 1C). These results suggest that in the absence of Notch activity, actin cytoskeleton rearrangement in PE precursor cells is impaired, and as a result, PE size is reduced.

We next sought to test whether overactivation of the Notch pathway would induce the reciprocal phenotype. To this end, we used the transgenic line *UAS:NICD‐myc* ^*KCA3*^,^28^ which drives the expression of the intracellular domain of the Notch receptor (NICD) under the *UAS* promoter. When combined with a *Gal4* transgene, this line allows overexpressing *NICD* in specific tissues or cell populations. To determine the cell type in which the activity of NICD might be needed to influence PE formation, we crossed *UAS:NICD‐myc* ^*KCA3*^ transgenic fish with *wt1b(BAC):Gal4FF* animals to drive *NICD* expression in the DP and PE, or with *fli1a:gal4* ^*ubs3Tg*^,^29^ to overexpress *NICD* only in endothelial and endocardial cells (Figure 1D). To determine the effect of Notch gain of function on PE formation, we performed these experiments in animals carrying the *epi:GFP* transgene. Whereas activation of the Notch pathway in *wt1b* ^+^ cells did not affect PE formation, we found that overexpression of *NICD* in endothelial cells resulted in a significant increase in PE cell numbers (13 ± 4 cells in *fli1a* ^+^ vs. 8 ± 3 cells in nontransgenic zebrafish) (Figure 1E).

Overall, our findings demonstrate that activation of the Notch signaling pathway in endothelial cells increased PE size, which suggests that paracrine signals from the underlying endothelium and endocardium may guide PE formation.

### 2.2 Notch signaling acts on endothelial cells

**FIGURE 2:**
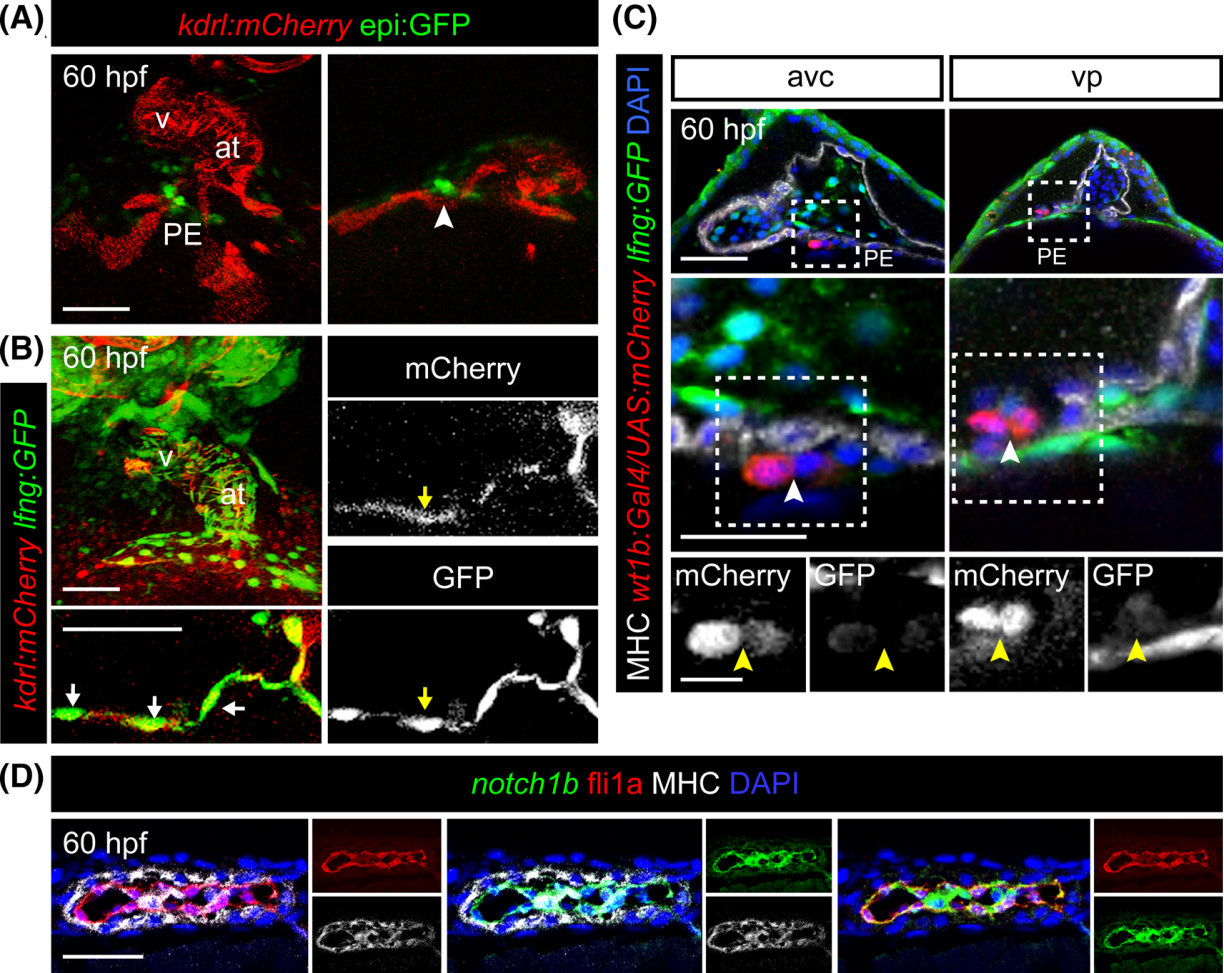
Notch signaling is active in endothelial cells. A, 3D projection and optical section of a 60 hpf *epi:GFP*; *kdrl:mCherry* double transgenic embryos. GFP^+^ pericardium and PE are shown in green, the mCherry^+^ endothelium is in red. Arrowhead, PE cluster. B, Maximum projection and optical sections of a 60 hpf zebrafish heart. *lnfg:GFP* ^+^ cells (green) colocalize with the endogenous mCherry^+^ endothelium (red) of *kdrl:mCherry*. Arrows, *lnfg:GFP* ^+^/*kdrl:mCherry* ^+^ cells. C, Optical sections through the avc and vp heart regions of 60 hpf *wt1b:Gal4*;*UAS:mCherry*; *lnfg:GFP* embryos immunostained for mCherry (red), GFP (green), myosin heavy chain (MHC, white) and nuclei counterstained with DAPI (blue). Zoomed views including single channels are shown below. Arrowheads, PE cluster. D, Fluorescent in situ mRNA hybridization on a 60 hpf *fli1a*:GFP ventricular heart section with *notch1b* riboprobe (green) followed by immunofluorescence for GFP (red) and myosin heavy chain (MHC, gray). Nuclei are counterstained with DAPI (blue). at, atrium; avc, atrioventricular canal; DP, dorsal pericardium; hpf; hours post fertilization; PE, proepicardium; v, ventricle; vp, venous pole. Scale bar: 50 μm (25 μm in C middle images and in D; 10 μm in C zoomed images)

Our results predict that manipulating Notch signaling in the endothelium influences PE formation. Accordingly, we observed that the PE forms right over *kdrl*:mCherry^+^ endocardial precursor cells located at the cardiac inflow tract (Figure 2A). To determine whether Notch signaling is active in this cell population, we examined *kdrl:mCherry*; *ET33‐mi60a* animals in which the endothelium is marked by mCherry expression and expression of GFP is driven by regulatory sequences of the Notch target *lunatic fringe* (*lfng*).^30^, ^31^ We detected *lfng*:GFP expression in endothelial cells of the inflow tract of the forming heart tube, suggesting that these cells are Notch responsive (Figure 2B). In PE cells, marked with *wt1b:Gal4;UAS*:mCherry expression, we were not able to detect high *lnfg*:GFP expression (Figure 2C). Thus, Notch activity seems to be restricted to the endothelium/endocardium and not present in PE cells. Fluorescent in situ hybridization also revealed that *notch1b* colocalizes with endocardial *fli1a*:GFP^+^ cells, but not with the myocardial cells immunostained with anti‐myosin heavy chain (MHC) (Figure 2D). Taken together, our results show that Notch signaling acts within the endothelium/endocardium and not on PE cells for PE formation.

### 2.3 Notch activation rescues proepicardium formation upon Myosin‐II inhibition

**FIGURE 3:**
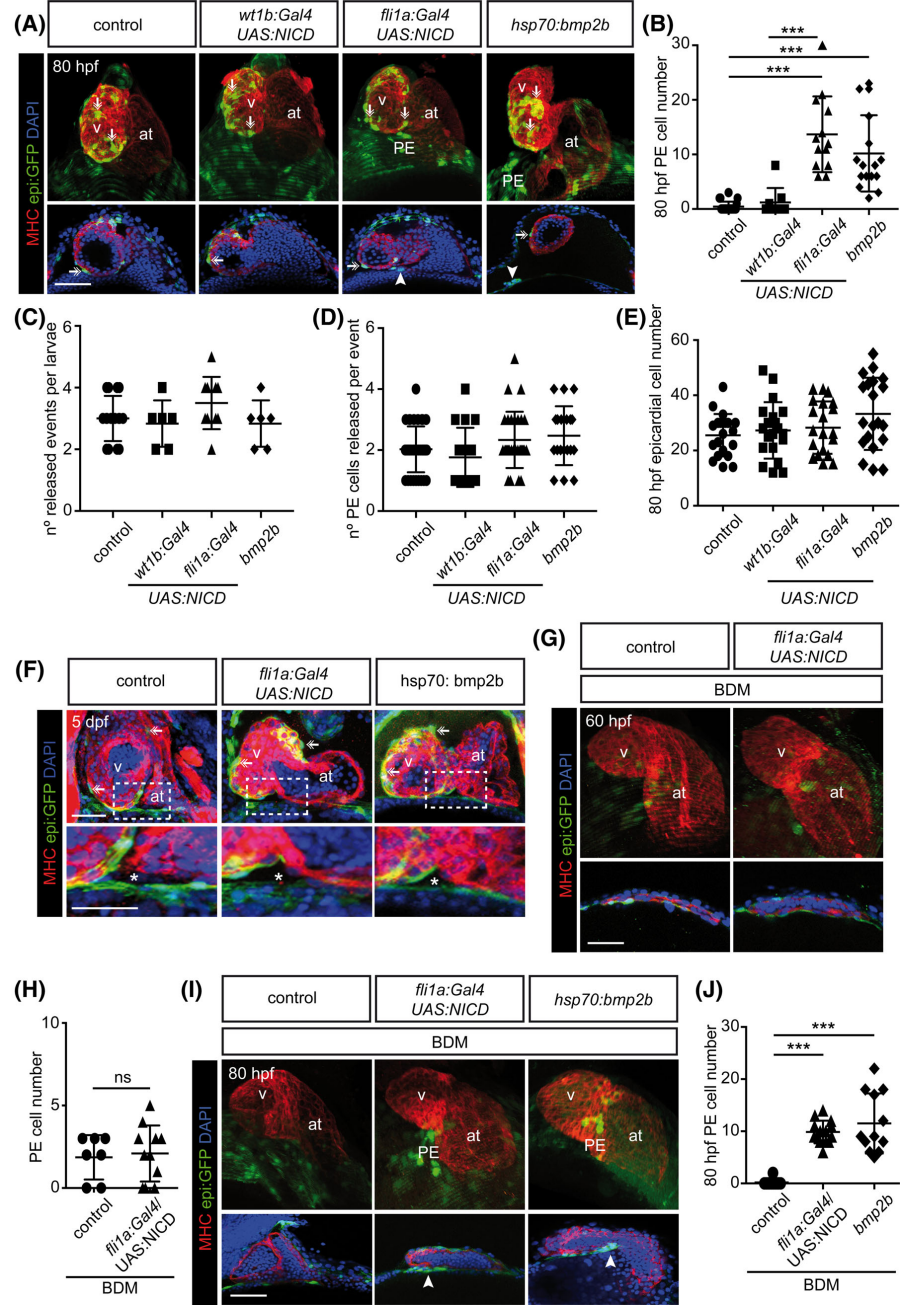
Notch signaling rescues proepicardium formation upon Myosin‐II inhibition at 80 hpf. A, E, and G, *epi:GFP* embryos immunostained for GFP (green), myosin heavy chain (MHC, red) and nuclei counterstained with DAPI (blue). Top panels, 3D projections and lower panels optical sections. The DP was digitally isolated in 3D projections. A, 80 hpf control zebrafish heart compared with those overexpressing *NICD* in pericardial and PE cells (*wt1b:Gal4*), endothelial cells (*fli1a:Gal4*) or *bmp2b* overexpressing embryos. Arrowheads, PE cluster. Arrows, epicardial cells. B, Quantification of PE cell number in A. C, Number of PE release events per larvae observed from 58 to 65 hpf. D, Number of PE cells released per event of cell release per larvae from 58 to 65 hpf. E, Quantification of epicardial cell number in A. F, Top panels, maximal projection of three optical sections and zoomed views are shown below. 5 days post fertilization (5 dpf) control zebrafish heart compared with those overexpressing *NICD* in endothelial cells (*fli1a:Gal4*) or *bmp2b* overexpressing embryos. Asterisk mark the region of PE formation at 60 hpf. Arrows, epicardial cells. G, 60 hpf BDM‐treated control zebrafish heart and heart from embryo overexpressing *NICD* in fli1a^+^ endothelial cells. H, Quantification of PE cell number of conditions as shown in G. I, 80 hpf BDM‐treated control heart, heart from embryo overexpressing *NICD* in fli1a^+^ endothelial cells and heart from an embryo overexpressing *bmp2b*. Arrowheads, PE cluster. J, Quantification of PE cell number of conditions shown in I. at, atrium; BDM, 2,3‐butanedione monoxime; DP, dorsal pericardium; hpf; hours post fertilization; PE, proepicardium; v, ventricle. Scale bars: 50 μm. Data are means ± SD, one‐way ANOVA followed by Kruskal‐Wallis significant difference test, unpaired two‐tailed Student's *t*‐test in H. ***P < .001, ns, nonsignificant

We next aimed to test whether experimental activation of the Notch signaling pathway was sufficient to promote the formation of PE clusters at later stages of development. In control animals, PE clusters appear around 60 hpf but are undetectable at 80 hpf when epicardial cells start to colonize the myocardial surface (Figure 3A). In agreement with our previous results, overexpression of *NICD* using the EPDCs driver *wt1b:Gal4* failed to increase the number of PE clusters at 80 hpf. In contrast, *NICD* overexpression in *fli1a* ^+^ endothelial cells still enhanced PE cluster formation at 80 hpf (14 ± 8 PE cells in *fli1a* ^+^ vs. 1 ± 3 cells in *wt1b* ^+^ animals; *P* < .0001) (Figure 3A,B). We previously described, that overexpression of *bmp2b*, by using the *Tg(hsp70l:bmp2b)* ^fr13^ line and performing heat shock pulses at 26, 35 and 48 hpf, stimulated PE formation at 60 hpf^13^. Here we found that similar to *NICD* overexpression, it also resulted in PE clusters maintenance at 80 hpf (Figure 3A,B). One possible explanation for the increase in PE cluster size observed upon *NICD* or *bmp2b* overexpression might be a change in the cell release process from the PE into the pericardial cavity. We counted the number of PE cell release events during 8hr in vivo acquisitions but did not observe any differences between control, *NICD* or *bmp2b*‐overexpressing animals (Figure 3C). Similarly, the number of cells released per event did not differ between groups (Figure 3D). We also analyzed the impact of *NICD* and *bmp2b* overexpression on epicardium formation. Epicardial cell numbers were unaffected at 80 hpf as compared with untreated animals (Figure 3E). Similarly, we wanted to assess whether PE formation was stable over longer times, and we found that at 5 days post fertilization (dpf) the PE was no longer present (Figure 3F).

Actomyosin cytoskeleton dynamics are necessary for PE formation.^13^ The myosin II inhibitory drug 2,3‐butanedione monoxime (BDM) impairs PE formation in a reversible way.^14^ We previously showed that overexpression of *bmp2b* rescues PE cluster formation at 60 hpf upon BDM treatment.^13^ Thus, we sought to assess whether *NICD* overexpression was sufficient to rescue PE formation under BDM treatment. Contrary to *bmp2b* overexpression, PE formation was not rescued by *NICD* overexpression in *fli1a* ^+^ endothelial cells at 60 hpf (Figure 3G,H). However, at 80 hpf, PE clusters were observed in BDM‐treated animals that overexpressed Notch in *fli1a* ^+^ cells (10 ± 2 cells vs. 0 ± 1 cells in BDM only; *P* < .0001; (Figure 3I,J). *bmp2b* overexpression also allowed PE maintenance at 80 hpf under BDM treatment. Thus, while ectopic *bmp* overexpression can rescue PE formation at 60 hpf, Notch overexpression rescues PE formation with a certain delay, at 80 hpf.

### 2.4 Notch signaling acts upstream of Bmp pathway to control proepicardium formation

Given that Bmp2b is able to rescue the formation of the PE upon BDM treatment at 60 hpf,^13^ and that PE formation requires more time to recover by *NICD* overexpression (observed at 80 hpf but not 60 hpf), we hypothesize that Notch signaling may function upstream of Bmp pathway activation to control PE formation.

**FIGURE 4:**
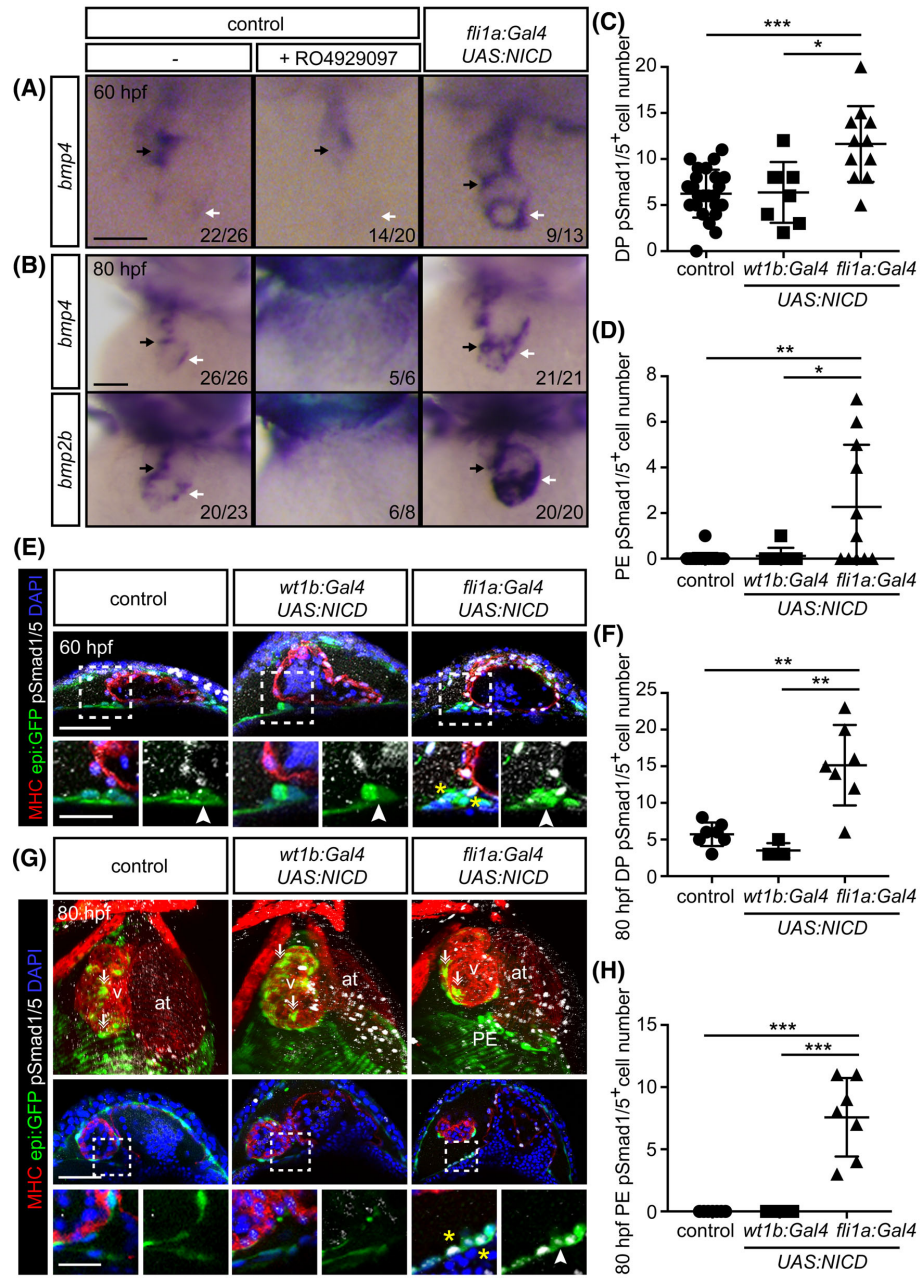
Endothelial Notch signaling enhances cardiac Bmp2/4 expression levels and induces pSmad 1/5 in the dorsal pericardium and proepicardium. A and B, Whole‐mount in situ hybridization for *bmp4* or *bmp2b* in control, RO‐treated and animals overexpressing *NICD* in endothelial cells (*fli1a:Gal4*). A, 60 hpf. B, 80 hpf. White arrows, venous pole. Black arrows, atrioventricular canal of the heart tube. C, Quantification of pSmad 1/5^+^ DP cell numbers at 60 hpf. D, Quantification of pSmad1/5^+^ PE cell numbers at 60 hpf. E, *epi:GFP* embryos immunostained for GFP (green), myosin heavy chain (MHC, red), pSmad1/5 (white) and nuclei counterstained with DAPI (blue). Top panels, optical sections of a 60 hpf control zebrafish heart compared with hearts from zebrafish overexpressing *NICD* in pericardial and PE cells (*wt1b:Gal4*) or in endothelial cells (*fli1a:Gal4*). Zoomed views are shown below. Arrowheads, PE cluster. Yellow asterisks, PE pSmad1/5^+^ cells. F, Quantification of DP pSmad1/5^+^ cell number at 80 hpf. G, 3D projections, optical sections and zoomed images of 80 hpf control zebrafish heart compared to those overexpressing *NICD* in pericardial and proepicardial cells (*wt1b:Gal4*) or in endothelial cells (*fli1a:Gal4*). The DP was digitally isolated in 3D projections. Arrowheads, PE cluster. Arrows, epicardial cells. Yellow asterisks, pSmad1/5^+^ PE cells. H, Quantification of PE pSmad 1/5^+^ cell number at 80 hpf. at, atrium; DP, dorsal pericardium; hpf; hours post fertilization; PE, proepicardium; v, ventricle. Scale bar: 50 μm (25 μm in zoomed images E, G). Data in C, D, F, and H are means ± SD, one‐way ANOVA followed by Kruskal‐Wallis significant difference test. *P < .05, **P < .01, ***P < .001

To determine whether these two pathways act coordinately during the formation of the PE, we first analyzed *bmp2/4* expression levels in the heart tube upon altering Notch signaling. Treatment with the Notch inhibitor RO from 48 hpf reduced heart tube *bmp4* expression levels at 60 hpf (n = 22/26 in control and 14/20 presented this phenotype in RO‐treated animals) (Figure 4A). In addition, the overexpression of *NICD* in *fli1a* ^*+*^ endothelial cells at 60 hpf increased *bmp4* levels in the heart tube (n = 9/13) (Figure 4A). When we overexpressed *NICD* in *fli1a* ^*+*^ cells at 80 hpf, *bmp4* as well as *bmp2b* expression levels increased in the heart (n = 21/21 and n = 20/20, respectively) (Figure 4B). On the contrary, RO‐treated animals revealed reduced *bmp4* and *bmp2b* expression levels at 80 hpf (Figure 4B).

To study if Notch‐induced augmented *bmp4/2b* in the heart results in increased Bmp signaling activation in PE cells, we first examined the presence of pSmad1/5^+^ DP and PE cells in *NICD* overexpressing animals. pSmad1/5 is a downstream effector of the Bmp pathway and its expression reflects the activation of the signaling pathway.^32^, ^33^ At 60 hpf, we observed that animals with ectopically activated Notch signaling in *fli1a* ^+^ cells, but not in *wt1b* ^+^ cells, exhibited more pSmad1/5^+^ DP and PE cells than controls at 60 hpf (Figure 4C–E) as well as at 80 hpf (Figure 4F–H).

**FIGURE 5:**
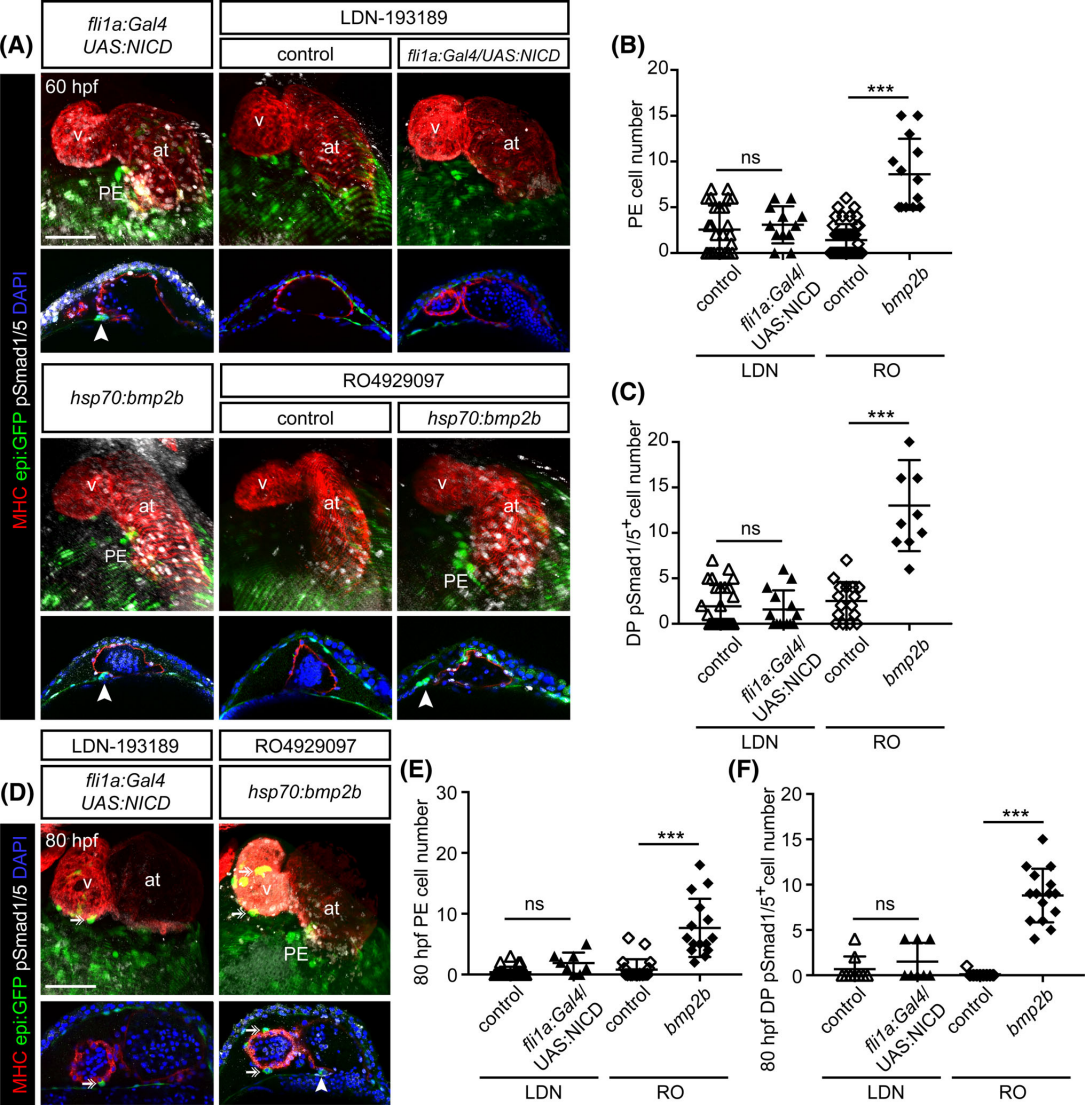
Endothelial Notch signaling acts upstream of Bmp to control PE formation. A and D, *epi:GFP* embryos at 60 hpf (A) or 80 hpf (D) either untreated or treated with LDN‐193189 or RO4929097 and immunostained for GFP (green), myosin heavy chain (MHC, red), pSmad1/5 (white) and nuclei counterstained with DAPI (blue). Top panels, 3D projections and lower panels optical sections. The DP was digitally isolated in 3D projections. Arrowhead, PE cluster. Arrows, epicardial cells. B, Quantification of PE cell number in A. C, Quantification of DP pSmad1/5^+^ cell number in A. E, Quantification of PE cell number in D. F, Quantification of DP pSmad1/5^+^ cell number in D. at, atrium; DP, dorsal pericardium; hpf, hours post fertilization; LDN, LDN‐193189; PE, proepicardium; RO, RO4929097; v, ventricle. DP digitally isolated in 3D projections. Scale bar: 50 μm. Data are means ± SD, unpaired two‐tailed Student's *t*‐test. ***P < .001, ns, nonsignificant

To further address the relationship between Notch and Bmp signaling pathways, we combined various Notch and Bmp gain‐ and loss‐of‐function scenarios. First, we assessed how Bmp inhibition influences Notch‐induced PE formation. To this end, we treated embryos overexpressing *NICD* in *fli1a* ^+^ cells with the Bmp inhibitor LDN‐193189^34^, ^35^ from 48 hpf onwards. LDN impairs PE formation, and *bmp2b* overexpression rescues the number of PE cells to a wild‐type situation.^13^ Here, we found that PE cluster formation was not rescued by *NICD* overexpression after LDN treatment. We counted 2 ± 3 cells in LDN treated vs. 3 ± 2 cells in LDN treated‐overexpressing *NICD* animals (Figure 5A,B); while the PE comprises 8 ± 3 cells in nontransgenic zebrafish (Figure 1C). We then tested the effect of overexpressing *bmp2b* upon Notch signaling inhibition with RO. We found that while 1 ± 2 cells comprised a PE in RO treated animals, in RO treated *bmp2b*‐overexpressing animals this number raised to 9 ± 4 cells (Figure 5A,B). Thus, *bmp2b* overexpression can override the negative impact of the lack of Notch signaling on the formation of the PE. Next, we evaluated how the different treatments affected pSmad1/5^+^ cell number in the DP. LDN treatment reduced the number of pSmad1/5^+^ cells in the DP, and *NICD* overexpression in *fli1a* ^+^ cells could not rescue the number of pSmad1/5^+^ cells upon LDN treatment (Figure 5C). Even at 80 hpf, *NICD* overexpression in *fli1a* ^+^ cells could neither rescue PE cluster formation nor the number of pSmad1/5^+^ cells upon LDN treatment (Figure 5D–F). On the contrary, the reduction in the number of PE cells and pSmad1/5^+^ cells upon RO administration could be recovered by *bmp2b* overexpression (Figure 5D–F).

Overall, we conclude that the Notch signaling pathway acts in the endothelium/endocardium to regulate Bmp expression in the heart tube, which is necessary for the formation of the PE.

## 3 DISCUSSION

We propose a model in which Notch activity in endothelial cells leads to the expression of *bmp2b* and *bmp4* in the heart tube, which subsequently signals to PE precursor cells promoting PE cluster formation.

PE and epicardium formation has been widely studied mainly in three animal species: the mouse, the chicken and the zebrafish. In the mouse and the chick, the PE is formed by a cauliflower‐like structure with a core of precardiac mesoderm and a mesothelial lining.^11^, ^12^ In the zebrafish, the PE is formed by small groups of mesothelial cells.^13^, ^14^, ^36^ In mice, PE cysts are thought to be released into the pericardial cavity,^37^ similar to what has been observed in the zebrafish.^14^ In the chick, the PE forms a bridge to the myocardium and PE cells are transferred in this way to the myocardial surface.^38^

Here, we studied the role of Notch on PE formation in the zebrafish and found some similarities but also species‐specific differences. While cell‐autonomous effects on PE formation and epicardium were previously found in mice,^22^, ^23^, ^24^ we did not observe enhanced PE formation when overexpressing *NICD* in PE cells using the *wt1b:Gal4* line. Notch receptors are expressed in the adult epicardium^39^ and in the endocardium,^40^, ^41^ suggesting that these two cardiac layers should be Notch responsive. It is possible that the *wt1b:Gal4* line is insufficient to robustly overexpress *NICD* in all PE cells and that this is the reason why we did not observe the same result as observed in the mouse. However, we clearly showed that overexpressing *NICD* in the endothelium is sufficient to enhance PE formation, indicating a non‐cell‐autonomous role for Notch signaling occurring in the zebrafish. The different tissue composition of the PE—being purely mesothelial in nature in the zebrafish, while harboring a mesoderm core in the mouse or chicken—might be one reason for the observed differences in Notch activity in the PE between these species.

BMP2 signaling has also been previously studied in the context of PE and epicardium formation, mostly in the chicken. There, BMP signaling has a concentration‐dependent effect on the specification of the precardiac mesoderm toward a PE or myocardial fate.^42^, ^43^ In the mouse, BMP signaling has been extensively studied in the context of epicardial EMT.^19^, ^44^, ^45^ In support to previous findings,^13^, ^16^ we confirmed that Bmp2b is required for PE cell extrusion during the time window of PE formation. BMP is also necessary to allow formation of PE extrusions and attachment of PE cells to the myocardial surface in chicken.^17^ In our previous article,^13^ we found that overexpression of *bmp2b* indeed led to an increase not only of PE formation but also of epicardialisation. At 60 hpf, hearts of fish overexpressing *bmp2b* revealed more epicardial cells than controls. However, here, at 80 hpf, we could not find differences in the amount of epicardial cell number in *bmp2b* overexpressing animals compared to controls. We suspect that 80 hpf is a time point too close to complete epicardialisation that would make it difficult to see differences. Also, once on the myocardial surface, compensatory mechanism such as less or more proliferation of epicardial cells might come into play.

The coordinated activity of NOTCH and BMP2 signaling during cardiovascular development has previously been studied in the mouse.^25^, ^26^ In this species, ectopic expression experiments indicate that myocardial BMP2 activates JAG1‐NOTCH1 signaling in the endocardium of the valve territory,^46^ while BMP2 signaling abrogation disrupts endocardial NOTCH signaling in this tissue.^25^ During the formation of the cardiac valve primordium, BMP2 and NOTCH1 act in concert to activate *Snail* expression and favors its nuclear localization that drive EMT. Also, ectopic *Notch1* expression throughout the myocardium represses *Bmp2* expression in this tissue. In cardiomyocytes, NOTCH activity has also been described in the myocardium, where it represses BMP signaling.^25^, ^47^ Consistently, blocking NOTCH signaling leads to the increase of BMP2 signaling in the PE, and NOTCH and BMP2 actively repress the formation of the PE.^23^ In zebrafish embryos, our results suggest an opposite mechanism, Notch signaling acts upstream of the Bmp pathway promoting PE formation and promote PE formation. Interestingly, an interaction between Notch and Bmp signaling has been reported during zebrafish cardiac regeneration. Upon genetic myocardium ablation endocardial Notch upregulation occurs, and subsequently induces *bmp10* expression in the myocardium.^48^ This is similar to what we observed in the context of embryonic development, suggesting that developmental mechanisms are reused during regeneration.

All these findings, and our own results on the interaction between Notch and Bmp signaling, show that the crosstalk between both pathways depends on the spatial‐temporal context, as well as on the species analyzed. Our finding also shows an example on how signaling arising from the luminal endothelium can influence the formation of the outermost cardiac layer, the epicardium. Collectively, our results reveal an example for the coordinated action of signaling molecules in controlling tissue morphogenesis across multiple tissue layers.

## 4 EXPERIMENTAL PROCEDURES

### 4.1 Zebrafish strains and husbandry

All experiments were approved by the Community of Madrid “Dirección General de Medio Ambiente” in Spain; and the “Amt für Landwirtschaft und Natur” from the Canton of Bern, Switzerland. Animals were housed and experiments performed in accordance with Spanish and Swiss bioethical regulations for the use of laboratory animals. Fish were maintained at a water temperature of 28°C. The following fish were used: wild‐type AB strain; *Et(−26.5Hsa.WT1‐gata2:EGFP)* ^cn1^ (*epi*:GFP)^14^; *Tg(hsp70l:bmp2b)* ^fr13^ ^49^; Tg(*uas::myc‐Notch1a‐intra*)^kca3^ ^28^; Tg(*fli1a:gal4*)^ubs3Tg^ ^29^; Tg(*kdrl::mCherry*)^ci5^ (from Elke Ober's laboratory), Et(*krt4:EGFP*)^sqet33mi60A^ ^50^; Tg(5x*UAS:mRFP*)^51^; Tg(*Tp1:CreERT2*)^s9^ ^51^, ^52^; Tg(*−3.5ubb:LOXP‐EGFP‐LOXP‐mCherry*) ^*cz1702*^ ^53^ and Tg(*fli1a:eGFP‐F*).^54^

### 4.2 Generation of the *TgBAC(wt1b:GAL4)cn18* transgenic line

The translational start codon of *wt1b* in the BAC clone CH73‐186G17 was replaced with the *galff‐polyA‐Kan* ^*R*^ cassette by Red/ET recombineering technology (GeneBridges) as described.^55^ To generate the targeting PCR product, *wt1b*‐specific primers were designed to contain 50 nucleotide homology arms around the ATG with ~20 nucleotide ends to amplify the *galff‐polyA‐Kan* ^*R*^ cassette. To facilitate transgenesis, the BAC‐derived *loxP* site was replaced with the *iTol2‐Amp* ^*R*^ cassette^56^ using the same technology. The final BAC was purified with the HiPure Midiprep kit (Invitrogen) and coinjected with Tol2 mRNA into *Tg(UAS:GFP)* embryos.^51^ The full name of this transgenic line is *TgBAC(wt1b:GALFF)*. Primers used to generate the *wt1b*‐GALFF targeting PCR product were the following:

wt1b_HA1_Gal4‐For: gacattttgaactcagatattctagtgttttgcaacccagaaaatccgtcaccATGAAGCTACTGTCTTCTATCGAAC.
wt1b_HA2‐KanR‐Rev: gcgctcaggtctctgacatccgatcccatcgggccgcacggctctgtcagTCAGAAGAACTCGTCAAGAA.
(lower case indicates homology arms).
The line has been deposited at ZFIN with the name *Tg(wt1b:Gal4)* ^*cn18*^.

### 4.3 Heat shock

Heat shock (HS) was performed on the embryos at 39°C in preheated water for 1 hr. When we overexpressed *bmp2b* by HS using *Tg(hsp70l:bmp2b)* ^fr13^ line HS was performed at 26, 35 and 48 hpf.

Animals treated with heat shock were genotyped after analysis. This allowed unbiased comparison and blinded quantification of experimental and control groups.

### 4.4 Immunofluorescence

Embryos were fixed overnight in 4% paraformaldehyde in PBS, washed in 0.01% PBS‐Tween‐20 (Sigma) and permeabilized with 0.5% Triton‐X100 (Sigma) in PBS for 20 min. Several washing steps were followed by 2 hr blocking with 5% goat serum, 5% BSA, 20 mM MgCl2 in PBS followed by overnight incubation with the primary antibody at 4°C. Secondary antibodies were diluted 1:500 in PBS and incubated for 3 hr. Nuclei were counterstained with DAPI (Invitrogen) for 30 min. Embryos were mounted in Vectashield (Vector).

Immunofluorescence staining on paraffin sections were performed as described in reference 57.

The antibodies and stains for immunofluorescence detection were as follows: anti‐myosin heavy chain, MHC, (MF20, ab_2147781 DSHB) at a 1:20 dilution, anti‐GFP (1010, Aveslab) at 1:1000, anti‐mCherry (Abcam) at 1:500 and anti‐pSmad1/5 (9516 Cell Signaling Technology) at 1:100, Phalloidin‐488 (A12379, Thermo Scientific). Secondary antibodies were the following: anti‐mouse IgG2b‐Alexa 568 (A21144, Thermo Scientific), anti‐chicken Alexa 488 (A11039, Thermo Scientific), anti‐rabbit Alexa 647 (A11036, Thermo Scientific), all diluted at 1:500.

Embryos were imaged with a Zeiss 780 confocal microscope fitted with a ×20 objective 1.0 NA with a dipping lens. Z‐stack images were acquired every 3–5 μm. Maximum intensity projections of images were 3D reconstructed in whole‐mount views using IMARIS software (Bitplane Scientific Software). The pericardial ventral tissue was digitally removed to provide a clearer view of the heart. Optical sections of 1–3 z‐slices were also reconstructed.

### 4.5 Quantification of DP and PE cells

PE cells have been described to emerge from two main regions of the DP: the avcPE appears close to the atrioventricular canal (avc), and the vpPE around the venous pole (vp). We counted each cell in each z plane using DAPI nuclear counterstain and GFP expression using the line *epi:GFP* as described.^13^ We took care not to count any cell twice. Cells with a round morphology at the vp or avc region were counted as PE cells, cells with a flat morphology in the DP were counted as DP cells.

### 4.6 Digital isolation of the dorsal pericardium

In *epi:GFP* embryos all pericardial cells express GFP. To visualize DP cells in 3D the VP was removed from all images of a Z‐stack. For single time‐point 3D reconstructions the surface function in Imaris was used and a mask was created to isolate the DP digitally.

### 4.7 Pharmacological treatments

Embryos were manually dechorionated and incubated with compounds from 48 hpf onwards. The following compounds were used: BDM (20 mM; Sigma), LDN‐193189 (20 μM; Sigma), RO4929097 (10 μM; Selleckchem).

### 4.8 In situ hybridization

ISH on whole‐mount embryos was performed as described^58^ using riboprobes against full coding sequence of *bmp4* or *bmp2b* cDNAs. Embryos at 60 hpf or 80 hpf were fixed in 4% PFA overnight, dehydrated in methanol series and stored at −20°C until its use. On day 1, embryos were bleached in 1.5% of H_2_O_2_ in methanol, rehydrated, washed in TBS with 0.1% Tween20 (TBST), digested with proteinase K 10 μg mL^−1^ for 17 min, rinsed in TBST, blocked the endogenous alkaline phosphatase with triethanolamine 0,1 M pH 8 with 0.25% of acetic anhydride for 20 min, washed in TBST, refixed in 4% PFA for 20 min. After washing again in TBST, embryos were prehybridized at 68°C for at least 1 hr. The antisense riboprobe was added at 0.5 μg mL^−1^. After overnight hybridization, two washes with 50% Formamide/5xSSC plus 2% Tween20 and four washes with 2xSSC plus 0.2% Tween20, all at 68°C were performed. Then, embryos were transferred to RT, washed in TBST and incubated with 10% heat inactivated goat serum, 1.2% of blocking reagent (Roche, 11096176001) in maleic acid buffer (MABT). Then, embryos were incubated overnight with 1:4000 dilution of anti‐digoxigenin‐AP antibody (Roche, 11093274910) in blocking solution. After overnight incubation, embryos were washed in MABT and developed in BM‐Purple until signal was detected.

Fluorescent in situ hybridization were performed using riboprobe against full coding sequence of *notch1b* cDNAs^59^ combined with immunostaining on paraffin sections. Sections were deparaffinized, postfixed 20 min with PFA 4%, washed with PBS, treated with proteinase K 10 μm mL^−1^ for 10 min at 37°C, washed with PBS, postfixed with PFA 4% for 5 min, washed with PBS, treated with HCl 0.07 N for 15 min, washed with PBS, treated with 0.25% acetic anhydride in triethanolamine 0.1 M pH 8 for 10 min, washed with PBS, washed with RNAse free water and then hybridized with the probe in prehybridization buffer overnight at 65°C. The following day sections where washed twice with posthybridization buffer 1 (50% Formamide, 5xSSC, 1% SDS) for 30 min at 65°C and twice with posthybridization buffer 2 (50% Formamide, 2xSSC, 1% SDS). Then, sections were washed with MABT buffer at room temperature, and incubated at least 2 hr in blocking solution at room temperature. They were next incubated overnight with anti‐digoxigenin‐POD antibody (1:500) in blocking solution. The third day, they were washed with MABT for several hours, after that sections were incubated with 1:200 tyramides (Perkin‐Elmer, NEL701A001KT), washed with PBST. Afterwards, immunofluorescence on sections was performed using anti‐GFP and anti‐myosin heavy chain (MHC) antibodies (overnight incubation at 4°C) and followed by PBS 0.1% Tween20 washes and incubation with secondary antibodies (as described above). After a second round of PBS 0.1% Tween20 washes sections were counterstained with DAPI.

### 4.9 Statistical analysis

Student's unpaired *t*‐test for comparisons between two groups or one‐way ANOVA analysis of variance for comparisons between more than two groups was used when normal distribution could be assumed. When the normality assumption could not be verified with a reliable method, the Kruskal‐Wallis test was used. Model assumptions of normality and homogeneity were checked with conventional residual plots. The specific test used in each comparison is indicated in the figure legend. Calculations were made with Microsoft Excel and GraphPad. *P*‐values are indicated either in the figure legends or the main text or summarized.

Raw Data leading to the Figures of this article has been deposited in Mendeley under DOI: 10.17632/g9xgv9s7hn.1.

## AUTHOR CONTRIBUTIONS

**Laura Andrés‐Delgado:** Conceptualization; formal analysis; funding acquisition; investigation; methodology; validation; visualization; writing‐original draft; writing‐review and editing. **Maria Galardi‐Castilla:** Investigation; methodology; writing‐review and editing. **Juliane Munch:** Investigation; methodology; writing‐review and editing. **Marina Peralta:** Methodology; writing‐review and editing. **Alexander Ernst:** Investigation; validation; writing‐review and editing. **Juan Manuel González‐Rosa:** Resources; writing‐review and editing. **Federico Tessadori:** Resources; writing‐review and editing. **Luis Santamaría:** Supervision; writing‐review and editing. **Jeroen Bakkers:** Resources; supervision; writing‐review and editing. **Julien Vermot:** Funding acquisition; supervision; writing‐review and editing. **José Luis de la Pompa:** Funding acquisition; supervision; writing‐review and editing. **Nadia Mercader:** Conceptualization; funding acquisition; project administration; supervision; validation; writing‐original draft; writing‐review and editing.

## References

[1] Olivey HE , Svensson EC . Epicardial‐myocardial signaling directing coronary vasculogenesis. Circ Res. 2010;106(5):818‐832. 20299672 10.1161/CIRCRESAHA.109.209197 PMC2843003

[2] Perez‐Pomares JM , de la Pompa JL . Signaling during epicardium and coronary vessel development. Circ Res. 2011;109(12):1429‐1442. 22158650 10.1161/CIRCRESAHA.111.245589

[3] Weinberger M , Simões FC , Patient R , Sauka‐Spengler T , Riley PR . Functional heterogeneity within the developing zebrafish epicardium. Dev Cell. 2020;52(5):574‐590. e576. 32084358 10.1016/j.devcel.2020.01.023 PMC7063573

[4] Chau YY , Bandiera R , Serrels A , et al. Visceral and subcutaneous fat have different origins and evidence supports a mesothelial source. Nat Cell Biol. 2014;16(4):367‐375. 24609269 10.1038/ncb2922 PMC4060514

[5] Liu Q , Huang X , Oh JH , et al. Epicardium‐to‐fat transition in injured heart. Cell Res. 2014;24(11):1367‐1369. 25257468 10.1038/cr.2014.125 PMC4220154

[6] Yamaguchi Y , Cavallero S , Patterson M , et al. Adipogenesis and epicardial adipose tissue: a novel fate of the epicardium induced by mesenchymal transformation and PPARγ activation. Proc Natl Acad Sci U S A. 2015;112(7):2070‐2075. 25646471 10.1073/pnas.1417232112 PMC4343131

[7] Kikuchi K , Gupta V , Wang J , et al. tcf21+ epicardial cells adopt non‐myocardial fates during zebrafish heart development and regeneration. Development. 2011;138(14):2895‐2902. 21653610 10.1242/dev.067041 PMC3119303

[8] Männer J . Does the subepicardial mesenchyme contribute myocardioblasts to the myocardium of the chick embryo heart? A quail‐chick chimera study tracing the fate of the epicardial primordium. Anat Rec. 1999;255(2):212‐226. 10359522 10.1002/(sici)1097-0185(19990601)255:2<212::aid-ar11>3.3.co;2-o

[9] Swonger JM , Liu JS , Ivey MJ , Tallquist MD . Genetic tools for identifying and manipulating fibroblasts in the mouse. Differentiation. 2016;92(3):66‐83. 27342817 10.1016/j.diff.2016.05.009 PMC5079827

[10] Kennedy‐Lydon T , Rosenthal N . Cardiac regeneration: epicardial mediated repair. Proc Biol Sci. 2015;282(1821):20152147. 26702046 10.1098/rspb.2015.2147 PMC4707759

[11] Maya‐Ramos L , Cleland J , Bressan M , Mikawa T . Induction of the proepicardium. J Dev Biol. 2013;1(2):82‐91. 23956959 10.3390/jdb1020082 PMC3744371

[12] Schulte I , Schlueter J , Abu‐Issa R , Brand T , Manner J . Morphological and molecular left‐right asymmetries in the development of the proepicardium: a comparative analysis on mouse and chick embryos. Dev Dyn. 2007;236(3):684‐695. 17238175 10.1002/dvdy.21065

[13] Andrés‐Delgado L , Ernst A , Galardi‐Castilla M , et al. Actin dynamics and the Bmp pathway drive apical extrusion of proepicardial cells. Development. 2019;146(13):dev174961. 31175121 10.1242/dev.174961 PMC6633599

[14] Peralta M , Steed E , Harlepp S , et al. Heartbeat‐driven pericardiac fluid forces contribute to epicardium morphogenesis. Curr Biol. 2013;23(18):1726‐1735. 23954432 10.1016/j.cub.2013.07.005

[15] Plavicki JS , Hofsteen P , Yue MS , Lanham KA , Peterson RE , Heideman W . Multiple modes of proepicardial cell migration require heartbeat. BMC Dev Biol. 2014;14:18. 24885804 10.1186/1471-213X-14-18 PMC4048602

[16] Liu J , Stainier DY . Tbx5 and Bmp signaling are essential for proepicardium specification in zebrafish. Circ Res. 2010;106(12):1818‐1828. 20413782 10.1161/CIRCRESAHA.110.217950 PMC2892550

[17] Ishii Y , Garriock RJ , Navetta AM , Coughlin LE , Mikawa T . BMP signals promote proepicardial protrusion necessary for recruitment of coronary vessel and epicardial progenitors to the heart. Dev Cell. 2010;19(2):307‐316. 20708592 10.1016/j.devcel.2010.07.017 PMC2925255

[18] Iyer D , Gambardella L , Bernard WG , et al. Robust derivation of epicardium and its differentiated smooth muscle cell progeny from human pluripotent stem cells. Development. 2016;143(5):904. 26932673 10.1242/dev.136143 PMC4813345

[19] Witty AD , Mihic A , Tam RY , et al. Generation of the epicardial lineage from human pluripotent stem cells. Nat Biotechnol. 2014;32(10):1026‐1035. 25240927 10.1038/nbt.3002 PMC4192149

[20] MacGrogan D , Munch J , de la Pompa JL . Notch and interacting signalling pathways in cardiac development, disease, and regeneration. Nat Rev Cardiol. 2018;15(11):685‐704. 30287945 10.1038/s41569-018-0100-2

[21] Tetzlaff F , Fischer A . Control of blood vessel formation by Notch signaling. Adv Exp Med Biol. 2018;1066:319‐338. 30030834 10.1007/978-3-319-89512-3_16

[22] Yang K , Doughman YQ , Karunamuni G , et al. Expression of active Notch1 in avian coronary development. Dev Dyn. 2009;238(1):162‐170. 19097050 10.1002/dvdy.21811 PMC2929638

[23] del Monte G , Casanova JC , Guadix JA , et al. Differential Notch signaling in the epicardium is required for cardiac inflow development and coronary vessel morphogenesis. Circ Res. 2011;108(7):824‐836. 21311046 10.1161/CIRCRESAHA.110.229062

[24] Grieskamp T , Rudat C , Ludtke TH , Norden J , Kispert A . Notch signaling regulates smooth muscle differentiation of epicardium‐derived cells. Circ Res. 2011;108(7):813‐823. 21252157 10.1161/CIRCRESAHA.110.228809

[25] Luna‐Zurita L , Prados B , Grego‐Bessa J , et al. Integration of a Notch‐dependent mesenchymal gene program and Bmp2‐driven cell invasiveness regulates murine cardiac valve formation. J Clin Invest. 2010;120(10):3493‐3507. 20890042 10.1172/JCI42666 PMC2947227

[26] Wang Y , Wu B , Chamberlain AA , et al. Endocardial to myocardial notch‐wnt‐bmp axis regulates early heart valve development. PLoS One. 2013;8(4):e60244. 23560082 10.1371/journal.pone.0060244 PMC3613384

[27] Conner C , Ackerman KM , Lahne M , Hobgood JS , Hyde DR . Repressing notch signaling and expressing TNFalpha are sufficient to mimic retinal regeneration by inducing Muller glial proliferation to generate committed progenitor cells. J Neurosci. 2014;34(43):14403‐14419. 25339752 10.1523/JNEUROSCI.0498-14.2014 PMC4205560

[28] Scheer N , Riedl I , Warren JT , Kuwada JY , Campos‐Ortega JA . A quantitative analysis of the kinetics of Gal4 activator and effector gene expression in the zebrafish. Mech Dev. 2002;112(1–2):9‐14. 11850174 10.1016/s0925-4773(01)00621-9

[29] Herwig L , Blum Y , Krudewig A , et al. Distinct cellular mechanisms of blood vessel fusion in the zebrafish embryo. Curr Biol. 2011;21(22):1942‐1948. 22079115 10.1016/j.cub.2011.10.016

[30] Bruckner K , Perez L , Clausen H , Cohen S . Glycosyltransferase activity of fringe modulates Notch‐Delta interactions. Nature. 2000;406(6794):411‐415. 10935637 10.1038/35019075

[31] Moloney DJ , Panin VM , Johnston SH , et al. Fringe is a glycosyltransferase that modifies Notch. Nature. 2000;406(6794):369‐375. 10935626 10.1038/35019000

[32] Massague J , Seoane J , Wotton D . Smad transcription factors. Genes Dev. 2005;19(23):2783‐2810. 16322555 10.1101/gad.1350705

[33] Wrighton KH , Lin X , Feng XH . Phospho‐control of TGF‐beta superfamily signaling. Cell Res. 2009;19(1):8‐20. 19114991 10.1038/cr.2008.327 PMC2929013

[34] Bjorklund O , Halldner‐Henriksson L , Yang J , et al. Decreased behavioral activation following caffeine, amphetamine and darkness in A3 adenosine receptor knock‐out mice. Physiol Behav. 2008;95(5):668‐676. 18930070 10.1016/j.physbeh.2008.09.018

[35] Cuny GD , Yu PB , Laha JK , et al. Structure–activity relationship study of bone morphogenetic protein (BMP) signaling inhibitors. Bioorg Med Chem Lett. 2008;18(15):4388‐4392. 18621530 10.1016/j.bmcl.2008.06.052 PMC2570262

[36] Serluca FC . Development of the proepicardial organ in the zebrafish. Dev Biol. 2008;315(1):18‐27. 18206866 10.1016/j.ydbio.2007.10.007

[37] Rodgers LS , Lalani S , Runyan RB , Camenisch TD . Differential growth and multicellular villi direct proepicardial translocation to the developing mouse heart. Dev Dyn. 2008;237(1):145‐152. 18058923 10.1002/dvdy.21378

[38] Nahirney PC , Mikawa T , Fischman DA . Evidence for an extracellular matrix bridge guiding proepicardial cell migration to the myocardium of chick embryos. Dev Dyn. 2003;227(4):511‐523. 12889060 10.1002/dvdy.10335

[39] Zhao L , Borikova AL , Ben‐Yair R , et al. Notch signaling regulates cardiomyocyte proliferation during zebrafish heart regeneration. Proc Natl Acad Sci U S A. 2014;111(4):1403‐1408. 24474765 10.1073/pnas.1311705111 PMC3910613

[40] Munch J , Grivas D , Gonzalez‐Rajal A , Torregrosa‐Carrion R , de la Pompa JL . Notch signalling restricts inflammation and serpine1 expression in the dynamic endocardium of the regenerating zebrafish heart. Development. 2017;144(8):1425‐1440. 28242613 10.1242/dev.143362

[41] Raya A , Koth CM , Buscher D , et al. Activation of Notch signaling pathway precedes heart regeneration in zebrafish. Proc Natl Acad Sci U S A. 2003;100(suppl 1):11889‐11895. 12909711 10.1073/pnas.1834204100 PMC304103

[42] Kruithof BP , van Wijk B , Somi S , et al. BMP and FGF regulate the differentiation of multipotential pericardial mesoderm into the myocardial or epicardial lineage. Dev Biol. 2006;295(2):507‐522. 16753139 10.1016/j.ydbio.2006.03.033

[43] van Wijk B , van den Berg G , Abu‐Issa R , et al. Epicardium and myocardium separate from a common precursor pool by crosstalk between bone morphogenetic protein‐ and fibroblast growth factor‐signaling pathways. Circ Res. 2009;105(5):431‐441. 19628790 10.1161/CIRCRESAHA.109.203083 PMC2861358

[44] Hill CR , Sanchez NS , Love JD , et al. BMP2 signals loss of epithelial character in epicardial cells but requires the Type III TGFβ receptor to promote invasion. Cell Signal. 2012;24(5):1012‐1022. 22237159 10.1016/j.cellsig.2011.12.022 PMC3288519

[45] Lockhart MM , Boukens BJ , Phelps AL , et al. Alk3 mediated Bmp signaling controls the contribution of epicardially derived cells to the tissues of the atrioventricular junction. Dev Biol. 2014;396(1):8‐18. 25300579 10.1016/j.ydbio.2014.09.031 PMC4252836

[46] Papoutsi T , Luna‐Zurita L , Prados B , Zaffran S , de la Pompa JL . Bmp2 and Notch cooperate to pattern the embryonic endocardium. Development. 2018;145(13):dev163378. 29853617 10.1242/dev.163378

[47] Luxan G , D'Amato G , MacGrogan D , de la Pompa JL . Endocardial Notch signaling in cardiac development and disease. Circ Res. 2016;118(1):e1‐e18. 26635389 10.1161/CIRCRESAHA.115.305350

[48] Galvez‐Santisteban M , Chen D , Zhang R , et al. Hemodynamic‐mediated endocardial signaling controls in vivo myocardial reprogramming. Elife. 2019;8:e44816. 10.7554/eLife.44816 PMC6592682 31237233

[49] Chocron S , Verhoeven MC , Rentzsch F , Hammerschmidt M , Bakkers J . Zebrafish Bmp4 regulates left‐right asymmetry at two distinct developmental time points. Dev Biol. 2007;305(2):577‐588. 17395172 10.1016/j.ydbio.2007.03.001

[50] Poon KL , Liebling M , Kondrychyn I , Garcia‐Lecea M , Korzh V . Zebrafish cardiac enhancer trap lines: new tools for in vivo studies of cardiovascular development and disease. Dev Dyn. 2010;239(3):914‐926. 20063419 10.1002/dvdy.22203

[51] Asakawa K , Suster ML , Mizusawa K , et al. Genetic dissection of neural circuits by Tol2 transposon‐mediated Gal4 gene and enhancer trapping in zebrafish. Proc Natl Acad Sci U S A. 2008;105(4):1255‐1260. 18202183 10.1073/pnas.0704963105 PMC2234125

[52] Ninov N , Hesselson D , Gut P , Zhou A , Fidelin K , Stainier DY . Metabolic regulation of cellular plasticity in the pancreas. Curr Biol. 2013;23(13):1242‐1250. 23791726 10.1016/j.cub.2013.05.037 PMC4206552

[53] Mosimann C , Zon LI . Advanced zebrafish transgenesis with Tol2 and application for Cre/lox recombination experiments. Methods Cell Biol. 2011;104:173‐194. 21924163 10.1016/B978-0-12-374814-0.00010-0

[54] Lawson ND , Weinstein BM . In vivo imaging of embryonic vascular development using transgenic zebrafish. Dev Biol. 2002;248(2):307‐318. 12167406 10.1006/dbio.2002.0711

[55] Bussmann J , Schulte‐Merker S . Rapid BAC selection for tol2‐mediated transgenesis in zebrafish. Development. 2011;138(19):4327‐4332. 21865323 10.1242/dev.068080

[56] Suster ML , Abe G , Schouw A , Kawakami K . Transposon‐mediated BAC transgenesis in zebrafish. Nat Protoc. 2011;6(12):1998‐2021. 22134125 10.1038/nprot.2011.416

[57] Gonzalez‐Rosa JM , Martin V , Peralta M , Torres M , Mercader N . Extensive scar formation and regression during heart regeneration after cryoinjury in zebrafish. Development. 2011;138(9):1663‐1674. 21429987 10.1242/dev.060897

[58] Jowett T , Lettice L . Whole‐mount in situ hybridizations on zebrafish embryos using a mixture of digoxigenin‐ and fluorescein‐labelled probes. Trends Genet. 1994;10(3):73‐74. 8178366 10.1016/0168-9525(94)90220-8

[59] Westin J , Lardelli M . Three novel Notch genes in zebrafish: implications for vertebrate Notch gene evolution and function. Dev Genes Evol. 1997;207(1):51‐63. 20607480 10.1007/s004270050091

